# Harmony in a panpsychist world

**DOI:** 10.1007/s11229-022-03974-7

**Published:** 2022-11-23

**Authors:** Bradford Saad

**Affiliations:** 1 Department of Philosophy and Religious Studies, Utrecht University, Utrecht, The Netherlands; 2 Sentience Institute, New York, US

**Keywords:** Panpsychism, Consciousness, Rationality, Psychophysical luck, Fine-tuning, Hedonic match, Functionalism, Multiverse, Design hypotheses, Teleological laws

## Abstract

Experiences tend to be followed by states for which they provide normative reasons. Such harmonious correlations cry out for explanation. Theories that answer or diminish these cries thereby achieve an advantage over theories that do neither. I argue that the main lines of response to these cries that are available to biological theorists—theorists who hold (roughly) that conscious subjects are generally biological entities—are problematic. And I argue that panpsychism—which holds (roughly) that conscious subjects are ubiquitous in nature—provides an attractive response to these explanatory cries. Taken together, these considerations underwrite a kind of ‘psychophysical fine-tuning’ argument in support of panpsychism, one that is reminiscent of cosmological fine-tuning arguments in favor of multiverse hypotheses.

## Introduction

We live in harmony. Pleasure is often followed by seeking behavior. Pain is often followed by avoidance behavior. Perceptual experiences often recommend judgments and actions that follow in their wake. Such harmonious correlations between experiences and states for which they provide reasons cry out for explanation. This paper develops an argument for the conclusion that such correlations support panpsychism, (roughly) the view that conscious subjects are ubiquitous in nature.^1^

The argument exploits a response to harmonious correlations that is available to panpsychism but not rival views. While panpsychism does not explain the harmonious correlations, it diminishes the extent to which they cry out for explanation by placing them within a larger body of psychophysical correlations, most of which are not harmonious. This response is readily available to panpsychists because they countenance minds outside the harmonious portion of the psychophysical nexus with which we are familiar. Rival theorists adopt a sparser conception of experiential reality that deprives them of this response. I’ll examine other responses that such theorists might adopt and explain why I find them unpersuasive.

While the argument aims to establish that harmonious correlations provide evidence for panpsychism, it is silent on how confident we should be in panpsychism. The answer to that question partly depends on the strength of other arguments for and against panpsychism. Evaluating those arguments is not on this paper’s agenda. So, I won’t be drawing any conclusions about whether panpsychism is true or how confident we should be in it.

Here’s the plan. Section 2 formulates panpsychism and rival views. Section 3 formulates the argument. Sections 4–8 defend its premises. Section 9 takes stock.

## Preliminaries

We can understand *panpsychism* more precisely as the view that consciousness is ubiquitous among physical^2^ entities. Given that biological entities constitute only a tiny minority of physical entities, panpsychism entails that the vast majority of conscious physical subjects are non-biological. But it leaves open what sorts of non-biological physical entities are conscious subjects. On some versions, the subjects in question are fundamental particles. On other versions, they include complex physical entities such as medium-sized dry goods or planets.

To a first approximation, *the biological view* holds that the vast majority of conscious physical subjects are biological. The biological view leaves open what sorts of biological entities are conscious subjects. For all the biological view says, the entities in question might be certain brains, central nervous systems, or animals. This formulation is compatible with *quasi-panpsychism*, the view that the vast majority of conscious physical subjects are biological and a thesis oddly like panpsychism is true. For instance, quasi-panpsychists might hold that the vast majority of conscious physical subjects are plants or neurons. Quasi-panpsychist views are rarely treated as live options.^3^ Since taking these views into account in what follows would distract from what I most wish to discuss, I will set quasi-panpsychism aside.^4^ To that end, I will officially understand the biological view as claiming that the vast majority of conscious physical entities are biological and denying quasi-panpsychism. My main conclusion, then, will be that harmonious correlations support panpsychism over the biological view.^5^

To preempt misunderstanding, I flag that panpsychism and the biological view are theses about the distribution of consciousness. They are silent on the nature of consciousness. Indeed, panpsychism and the biological view can each be combined with a physicalist, functionalist, dualist, or Russellian monist view about the nature of consciousness. And, as I have formulated it, panpsychism leaves open whether experiences of subjects like us can be explained in terms of the experiences of our more fundamental parts.

In what follows, I assume, contrary to some forms of idealism and substance dualism, that you and I are among the many conscious physical subjects in our universe. This is partly for convenience: substance dualists are invited to recast claims about physical subjects—including the claims of panpsychism and the biological view—as claims about subjects that are either themselves physical or else uniquely causally paired with physical objects.^6^ I suspect that the availability of idealist recasting will depend sensitively on what form of idealism one adopts.^7^ But I won’t explore the prospects for such recastings here.

## The argument

To formulate my argument more precisely, I need to say more about what harmonious correlations are. Harmonious correlations obtain between experiences, i.e. states that there is something it’s like to undergo, and states that they rationalize, i.e. provide normative reasons for. Such reasons need not be decisive. For our purposes, *pro tanto* reasons count as rationalizing the states for which they provide reasons no less than decisive reasons. Normative reasons will be understood broadly to include moral, epistemic, and prudential reasons. For simplicity, we can restrict our consideration of rationalized states to other experiences (e.g. occurrent judgments) and actions. Let’s say that a type of experience *E* figures in a *harmonious correlation* if, when subjects instantiate *E*, they often shortly thereafter enter a state *R* such that their having *E* rationalizes their entering *R*.

My argument can now be stated more precisely as follows:

- *Harmony*: Many experiential properties figure in harmonious correlations.
- *Urgency*: If true, Harmony cries out for explanation.
- *Conditional Support*: If Harmony cries out for explanation, it supports views that are better positioned to respond to its cries over other views.
- *Advantage*: Panpsychism is better positioned to respond to Harmony’s cries than the biological view.
- :. Harmony supports panpsychism over the biological view.

Below sections defend its premises.

## Harmony

Harmony’s truth can be appreciated by reflecting on three (partially overlapping) general classes of experiences. Each class plausibly contains enough types of experience that participate in harmonious correlations to make Harmony true.

First, there are *valenced experiences*. These include positive and negative somatic, olfactory, emotional, mood, and aesthetic experiences. When a subject undergoes a positively valenced experience, it gives her a reason to take actions that promote certain of its contents or to seek more experiences of that type. When a subject undergoes a negatively valenced experience, it gives her a reason to avoid having more experiences of that type or take actions against certain contents of her experience.^8^ It turns out that, upon having a valenced experience, subjects tend to exhibit seeking or avoidance behavior that is appropriate to the valence of their experience. Valenced features of experience come in degrees. For instance, experiences can be more or less joyous. The degree to which an experience exhibits positive or negative features influences what reasons it generates. For instance, if an experience includes both a mild pain and a severe pain, then it gives the subject a stronger reason to avoid the latter. We live in a world in which subjects do not merely behave in ways that are rationalized by the valence of positive and negative experiences. Their behavior also rationally aligns with more fine-grained facts about the degrees to which experiences exhibit positive and negative features.^9^

Second, there are *sensory experiences*. These include perceptual and imagery experiences. Such experiences give subjects reasons to have certain judgments and perform certain actions. If one has a visual experience as of a treasure chest, one has reason to judge “I’m having an experience as of a treasure chest”. And if one is on a quest for treasure, the experience recommends some actions (e.g. ones that are likely to open the chest, given that your experience is accurate) over others (e.g. those that are likely to destroy the chest’s contents). In our world, subjects frequently act and judge in the ways recommended by sensory experiences shortly after having them.

Finally, there are *cognitive experiences*. They plausibly include occurrent judgements, inner speech experiences, and perceptual experiences of language. Cognitive experiences can provide reasons for having subsequent cognitive experiences. For example, an auditory experience as of someone professing a desire for blackberries gives its subject a reason to judge that the speaker desires blackberries. Similarly, upon judging that Gary or Glenda committed the crime and that Glenda has an alibi, one has reason to judge that Gary committed the crime. Cognitive experiences can also provide reasons to perform actions. Upon judging that you want to win the chess match you are playing and that castling is your only way to avoid checkmate, your judgment gives you a reason to castle.

I should acknowledge that cognitive experiences are controversial in a number of respects.^10^ There is debate about whether they exist and about whether they constrain or determine contents of thought. I side with those who think that cognitive experiences exist and constrain thought (judgment) content. So, I take cognitive experiences to help make Harmony true. Those who doubt that cognitive experiences constrain thought contents may well doubt that such experiences help make Harmony true. Nonetheless, I claim that such theorists should accept Harmony in light of the correlations between non-cognitive sensory and valenced experiences and the states they rationalize.

In addition, there is debate about whether cognitive experiences are distinct from sensory experiences. Goff (2018) appeals to harmonious correlations in this debate. He uses them to challenge a view he calls *robust cognitive phenomenalism*, which holds that “[o]ccurrent thoughts are identical with, or constituted of, states of cognitive phenomenology… [and that] facts about consciousness are not grounded in functional facts” (*ibid*: 100) By ‘cognitive phenomenology’ Goff means phenomenology that is distinct from sensory phenomenology. In contrast, I use ‘cognitive experience’ in a way that does not definitionally entail non-sensory experience.

The gist of Goff’s challenge—which he dubs the ‘cognitive fine-tuning problem’—is that robust cognitive phenomenalists are at pains to explain why, on their view, cognitive experiences align with sensory experiences and functional facts in a way that respects norms of rationality. Goff’s challenge and my own differ in important respects.^11^ I think his challenge is misdirected. Robust cognitive phenomenalism’s commitment to cognitive experiences that are distinct from sensory experiences does not play an essential role in his argument. As a result, the cognitive fine-tuning problem evidently tells equally against one of robust cognitive phenomenalism’s competitors,^12^ namely the view that occurrent thoughts are constituted by a kind of sensory phenomenology.^13^ Moreover, in focusing on thought-involving harmonious correlations, his challenge neglects non-cognitive experiences—such as pains and pleasures—that are involved in harmonious correlations. Consequently, the cognitive fine-tuning problem for robust cognitive phenomenalism turns out to generalize far beyond its rather specific and controversial target. In contrast, my argument aims at a widely held and comparatively general target (the biological view).

## Harmony cries out for explanation

As Urgency claims, given that Harmony is true, it cries out for explanation. These cries trace to three facts.

First, experiences are not *normatively promiscuous*, they do not rationalize more or less any state—if they did, Harmony would be far less striking. Instead of being normatively promiscuous, experiences are *normatively selective*: a given experience would rationalize only a highly restricted class of states. If one randomly picked an experience and a state from our world, our nomic neighborhood, or the space of metaphysical possibility, one could reasonably be confident that the experience would not rationalize that state. Similarly, if one switched the experiences of any two randomly selected subjects in our world while holding their non-experiential states fixed, it is extremely likely that one would substantially diminish the extent to which their switched experiences participate in harmonious correlations.^14^

Second, *experience-state scarcity* obtains: at any given time, subjects have at most a small proportion of possible experiences and they are in only a small proportion of states, even with respect to the class of states that experiences can rationalize. If experience-state scarcity were false, we would not expect switching two subjects’ non-experiential states to substantially diminish the extent to which their experiences participate in harmonious correlations. Indeed, if experience-state scarcity were, false Harmony would be far less striking.

The fact that experiences are normatively selective and experience-state scarcity obtains suggests that if harmonious correlations are merely coincidences, then they are unlikely. This is not enough to show that Harmony is in need of special explanation. Unlikely mere coincidences happen. Indeed, in many cases, they are likely to happen. For instance, we expect there to be unlikely coincidences in lotteries between the winning number and a person’s ticket number.^15^ But not all seemingly unlikely correlations can be written off as mere coincidences. While a monkey’s typing a random-seeming string does not cry out for explanation, a monkey’s typing a sonnet does.^16^ This is so even if a randomly typing monkey has the same chance of producing the two strings. That’s because, given our background knowledge, a monkey’s typing a sonnet invites a non-chancy explanation (e.g. that monkeys have typed a vast number of strings), while a monkey’s typing a random-seeming string does not.^17^ Similarly, harmonious correlations are not random-seeming. And they invite a non-chancy explanation. Thus, when it comes to Harmony and Shakespearean monkeys, the explanation “something was bound to happen and it might as well have been this” doesn’t cut explanatory ice.

It may seem that Harmony admits of explanations that are available to almost everyone. One such explanation is causal: Harmony holds because experiences cause effects that they rationalize.^18^ Since we lack a worked out psychophysics, we don’t know exactly how experiences cause such effects. But we don’t need a worked out psychophysics to be confident that an explanation of this sort holds.

Of course, this sort of explanation is unavailable to epiphenomenalists who deny that experiences cause effects. But everyone, including epiphenomenalists, can accept a nomic explanation on which the laws of nature, in concert with initial conditions and normative principles, produce Harmony. After all, such posits would presumably explain the distribution of all states, including harmoniously correlated states. For this reason, it is a mistake to use harmonious correlations with the aim of posing a distinctive problem for epiphenomenalism.^19^ Admittedly, since we don’t know the exact character of these laws, conditions, and principles, we can’t say exactly how they lead to Harmony. Nonetheless, those of us who are antecedently committed to such entities can be confident that they do.

These explanations may be correct as far as they go. But it would be a mistake to infer that Harmony poses no explanatory difficulty for anyone who accepts them. Some responses to Harmony’s explanatory cries are better than others. There are two sorts of satisfactory responses to phenomena that cry out for explanation.

One simply gives a satisfactory explanation of the phenomena. What counts as a satisfactory explanation of phenomena that cry out for explanation? It will be enough for our purposes to identify two necessary conditions for such an explanation.^20^

First, on pain of vicious explanatory deferral, satisfactory explanations of phenomena that cry out for explanation must not invoke explanantia that emit explanatory cries with an urgency similar to that of the phenomena they are invoked to explain. Second, satisfactory explanations of phenomena that cry out for explanation need to be stable, i.e. they need to be such that, according to them, the explananda could not have easily failed to obtain.^21^ These conditions entail that a satisfactory explanation of Harmony needs to be urgency-reducing and stable. They also show why the above explanations are not (by themselves) enough to satisfactorily answer Harmony’s explanatory cries. Claiming that Harmony obtains because experiences cause effects that they rationalize would not by itself provide an explanation that meets these conditions. For some causes bring about effects in a precarious manner. And the question of why many types of experience cause states that they rationalize is no less urgent than the question of why Harmony obtains. Similarly, the question of why Harmony-underwriting laws, initial conditions, and normative principles co-obtain in a Harmony-inducing fashion is no less urgent than the question of why Harmony obtains.

The second sort of satisfactory response eliminates or substantially diminishes the extent to which the striking phenomena cry out for explanation. That the winning number matches Winslow’s lottery ticket cries out for explanation.^22^ Admittedly, the fact that many lottery tickets (with different numbers) were purchased does not answer those cries.

For, on some views, explanations require a causal connection between the explanans and the explanandum.^23^ It’s doubtful that there’s any such connection between the noted fact and Winslow’s winning. So, if explanation requires causal connection, it’s doubtful that that fact explains Winslow’s winning. Similarly, if explanations require counterfactual dependence between explanandum and explanans, it’s doubtful that many tickets being purchased explains Winslow's winning. Perhaps Winslow would still have won if his purchase were the only one. And perhaps there are nearby worlds in which many tickets are purchased but he doesn’t win.

Nonetheless, the fact that many tickets were purchased does reduce the extent to which Winslow’s winning cries out for explanation. If someone were initially puzzled by the match between Winslow’s ticket and the winning number, pointing out that many tickets were purchased would be a satisfactory response, even if not an explanation. My argument for Advantage is that a satisfactory response of this sort is available to panpsychists but not biological theorists (Sect. 6) and responses to Harmony that are available to biological theorists are unsatisfactory (Sect. 7).

None of this is to say that panpsychists should regard harmonious correlations as brute any more than it is to say that we should regard Winslow’s winning as brute. Winslow’s winning may be explained by his procedure for picking a number, the procedure for determining the winning number, and the fact that these procedures yielded the same number. This explanation would not be satisfactory on its own, as it appeals to a coincidence that is no less striking than the fact that it is invoked to explain. Still, it is preferable to taking Winslow’s winning as a brute fact. And that explanation gains appeal if we combine it with the urgency-reducing hypothesis that many tickets were purchased. Similarly, we have seen that harmonious correlations can be explained by appealing to laws that settle the distribution of experiences or their causal profiles. As with the proposed explanation of Winslow’s winning, these explanations of Harmony are not satisfactory on their own, as they are not urgency-reducing. Yet they are preferable to taking harmonious correlations as brute facts. And they gain appeal when combined with panpsychism if, as I will argue, panpsychism reduces the extent to which harmonious correlations call for explanation.

## How panpsychism can satisfactorily respond to harmony’s cries

One way to diminish the extent to which something cries out for explanation is to reveal it to be one outcome among many in a sample space that exhibits variation. Return to the monkey and lottery examples. That a monkey typed a sonnet cries out for explanation. These cries are diminished on the assumption that many monkeys have been typing and that nearly all the strings they produced were random-seeming. That Winslow’s ticket number matches the winning number cries out for explanation. These cries are diminished on the assumption that many people bought tickets, the vast majority of which had losing numbers. Similarly, I claim, the assumption that most types of experiences do not figure in harmonious correlations would substantially diminish the extent to which Harmony cries out for explanation.

This assumption is a natural one for panpsychists. They hold that relatively few conscious physical entities are biological. As biological theorists will agree, the only harmonious correlations that we know about involve experiences associated with organisms. Our best theories of non-biological physical entities do not seem to require their experiences (if such there be) to have states that figure in harmonious correlations. To suppose that such experiences figure in harmonious correlations would add nothing to the explanatory power of those theories or panpsychism. In fact, that supposition would diminish their explanatory power: while failing to pay any explanatory dues, harmonious correlations involving experiences of non-biological physical entities would cry out for explanation no less than harmonious correlations involving organisms’ experiences.

Where does this leave panpsychists? Evidently, it leaves them in the position of having no reason to posit harmonious correlations involving experiences of non-biological physical entities and having some reasons to reject such correlations. It is a short step from rejecting such correlations to holding that most experiential properties do not figure in harmonious correlations. To take it, panpsychists need only suppose that a relatively large portion of conscious physical entities—the non-biological ones—instantiates most of the experiential properties. The resulting panpsychist picture is one on which participating in harmonious correlations is the exception rather than the rule for experiences.^24^ That is, it is one on which the noted assumption that most types of experiences do not figure in harmonious correlations holds. Since that assumption substantially diminishes the extent to which Harmony cries out for explanation, I conclude that panpsychists are well-positioned to give a satisfactory response to Harmony’s explanatory cries.^25^

Could biological theorists co-opt this response by supposing that most types of experiences that biological entities have do not figure in harmonious correlations? Strictly speaking, this move is logically available. For instance, the assumption that most humans do not have pain experiences just before exhibiting pain-appropriate behavior might diminish the extent to which the fact that your pains tend to precede pain-appropriate behavior cries out for explanation. The trouble is that this seems to lead to skepticism about other biological minds. Of course, how such skepticism is to be avoided is a controversial matter. Nonetheless, it is an extremely plausible minimal requirement on avoiding such skepticism that we ascribe experiences to biological entities on the basis of their behavior in a way that places their experiences in harmonious correlations. Thus, given that skepticism about other biological minds is an unacceptable consequence, biological theorists cannot co-opt the panpsychist’s treatment of Harmony.

## Can biological theorists satisfactorily explain harmony?

I’ve argued that Harmony cries out for explanation and that panpsychists have a satisfactory response to these cries that is unavailable to biological theorists. But biological theorists can still escape my argument by providing another satisfactory response, one that justifies the rejection of Advantage. There are three forms that such an explanation might take. On the first, Harmony obtains in virtue of an explanatory relation running from experiences to states they rationalize. Section 7.1 considers explanations of this sort that appeal to teleological laws. On the second, Harmony obtains in virtue of an explanatory relation running from states that experiences rationalize to the experiences themselves. Section 7.2 considers functionalist explanations of this sort. On the third, Harmony obtains in virtue of an explanatory connection that runs from a common source to experiences and to states they rationalize. Section 7.3 considers an explanation of this sort that appeals to a designer.^26^

### Teleological laws

Why does Harmony hold? One way biological theorists might tackle this question is by arguing that experiences explain the subsequent states that they rationalize, thereby generating the harmonious correlations. But how might experiences explain states that they rationalize? Sect. 5 noted one possibility in passing: experiences explain such states by causing them. This suggestion requires development, as not just any sort of causal connection between experiences and states they rationalize will do. Causal relations that obtain only in virtue of non-normative features of their relata would leave biological theorists with a mystery as to why the causal and rationalizing powers of experiences align. In other words, at least without supplementation, the resulting explanation of Harmony would be unsatisfactory, as it would violate the urgency-reduction condition imposed in Sect. 5. To overcome this difficulty, biological theorists might claim that it is because experiences would rationalize certain states that they cause them.

This proposal can be put in terms of *teleological laws*, i.e. laws that mention normative phenomena. For instance, the following is a conceivable teleological law: for any type of experience *E*, if a subject instantiates *E* then *ceteris paribus E* will cause her to behave in the way her instantiating *E* most rationalizes. The idea would then be that a set of laws along these lines underwrites the harmonious correlations and in turn Harmony.

Such laws may strike some as relics of an outdated Aristotelian framework. But given a lightweight conception of *ceteris paribus* laws on which they are merely derivative entities, this reaction is unwarranted. If one conceives of *ceteris paribus* teleological laws as informative summaries of certain regularities that obtain in virtue of initial conditions and fundamental non-teleological laws, then it should be relatively uncontroversial that there are such laws that apply to biological subjects. Nonetheless, appealing to *ceteris paribus* teleological laws will not ultimately help biological theorists. The trouble is that there are presumably many metaphysically possible combinations of initial conditions and fundamental laws on the biological view that do not generate *ceteris paribus* teleological laws that explain Harmony. Thus, even granting that such laws in some sense explain Harmony on the biological view, they do not provide a satisfactory explanation. For appealing to them raises a similarly urgent explanandum: the obtaining of initial conditions and more fundamental non-teleological laws that explain *ceteris paribus* teleological laws that themselves explain Harmony. Nor is it clear that the proposal would yield a stable explanation of Harmony—one on which it would have been difficult for Harmony to fail to obtain—as a satisfactory explanation of Harmony requires. In fact, evidence against the stability of such an explanation can be found in cosmological fine-tuning, i.e. the existence of basic physical parameters that take values within narrow life-permitting ranges. For cosmological fine-tuning suggests that it easily could have been the case that parameters had life-forbidding values, values that would not have given rise to *ceteris paribus* teleological laws that explain Harmony on the biological view.

Here biological theorists might claim that experiences cause states that they rationalize in accordance with *fundamental* teleological laws, ones that do not derive from more basic laws.^27^ Since such laws would not derive from initial conditions and non-teleological laws, they would not inherit instability from adjustable parameters in the initial conditions or laws from which they arise. And since fundamental teleological laws would generate the correlations directly, rather than as a mysterious byproduct, they might seem to explain Harmony without violating the urgency-reduction condition.

While this bold proposal is initially attractive, it is also problematic on reflection. For it flouts a tempting and widely advocated constraint on theorizing about consciousness, namely that we should account for consciousness and its place in nature without positing violations of physical laws.^28^ This constraint is often manifest in discussions that motivate physicalist, epiphenomenalist, or Russellian monist views by noting that these views respect the causal closure of the physical domain. Thus, embracing the proposed explanation of Harmony would put biological theorists at odds with a tempting constraint and a wide range of views about consciousness.^29^ That said, this proposal to invoke fundamental teleological laws to explain Harmony may better cohere with interactionist dualism. Interactionist dualists are generally committed to fundamental psychophysical laws of some sort. And it’s not immediately obvious that such theorists should be averse to fundamental teleological laws.^30^ So, appealing to fundamental teleological laws to underwrite causal connections between experiences and states they rationalize and, in turn, explain Harmony at best seems like an option for biological theorists who are open to interactionist dualism.

But this option would be unattractive on some forms of interactionist dualism. In particular, it is an unattractive option on interactionist dualist views that construe the effects of experiences as causally overdetermined by experiences and non-experiential states. Granted, on such a view, biological theorists might satisfactorily explain Harmony by supposing that experience’s redundant contributions are underwritten by fundamental teleological laws. But the resulting theoretical package would require an additional non-teleological explanation of the harmonious correlations: given that the teleologically underwritten causal contributions are redundant, there will be a causal account of the harmonious correlations that makes no mention of such contributions. If such an account were true, it would raise an urgent explanatory question: why do experiences’ rationalizing and non-teleological causal powers align in a way that generates Harmony? It is not clear that this explanandum is less urgent than Harmony. Biological theorists who opt for overdetermination induced by a fundamental teleological law are thus at risk of replacing one explanatory problem with a similarly pressing one.

Fundamental teleological laws are perhaps best suited to explain Harmony on a radical form of interactionist dualism that denies that experiences’ causal contributions are redundant. On this view, experiences are non-physical states that produce effects that violate fundamental physical laws. This view is widely rejected because it bets against physics. A teleological version of this form of interactionist dualism would hold that experiences non-redundantly produce effects in accordance with fundamental teleological laws. Given suitable teleological laws, the view explains harmonious correlations as a consequence of experiences that, in accordance with those laws, cause effects that they rationalize. Moreover, unlike interactionist dualists who opt for overdetermination, theorists who accept this view need not countenance a non-teleological explanation of Harmony, much less one that raises similarly pressing explanatory difficulties. For they can hold that Harmony would have been false, had the teleological laws not held.

I think this is probably the best option for biological theorists who want to satisfactorily explain Harmony with teleological laws. Nonetheless, it faces problems. A relatively minor problem is that since the fundamental laws we know about seem to be non-teleological, uniformity considerations tell in favor of non-teleological interactionist laws over teleological interactionist laws. A more pressing problem is that it is far from clear that the view explains Harmony without violating the urgency-reduction condition. For we can still ask, why does the set of actually obtaining laws include Harmony-underwriting teleological ones, rather than any of the vast number of (teleological and non-teleological) psychophysical laws that wouldn’t have underwritten Harmony?^31^

To handle this problem, such theorists could appeal to a feature of such laws that makes them especially likely to obtain. The obvious candidate feature that comes to mind is that such laws would be favored by a designer.^32^ This would make the teleological explanation of Harmony parasitic on a design explanation. I defer consideration of design explanations until Sect. 7.3.

Let’s sum up. By positing teleological laws, biological theorists might explain why experiences cause states that they rationalize and, in turn, Harmony. However, to answer the argument, biological theorists would need to show that a *satisfactory* explanation of this sort is available. My attempts to spell out a teleological explanation of Harmony suggest that this need is not easily met. As we have seen, much of the difficulty traces to the urgency-reduction condition on providing a satisfactory explanation of Harmony.

### Functionalism

Rather than construe experiences as causes of states they rationalize, biological theorists could opt for a theory on which rationalized states help explain both the experiences that rationalize them and (in turn) Harmony. A natural choice for biological theorists looking to take this route is some form of *functionalism*, which holds that experiences obtain in virtue of functional facts. To see why functionalism does not provide a promising line of response to my argument, it will help to distinguish two versions of functionalism.

*Nomological functionalism* holds that experiences are non-physical states generated in accordance with contingent fundamental laws, and that the laws in question assign functional instantiation conditions to types of experience.^33^ For instance, a law of this sort might dictate that non-physical pains are instantiated whenever subjects enter states that are disposed to generate avoidance behavior. In contrast, *metaphysical functionalism* holds that subjects’ experiences essentially involve certain sorts of effects of those subjects’ states. The essential connection might or might not be a priori or conceptually necessary. Similarly, the essential connection might or might not be constitutive. Constitutive versions include: the role functionalist view that experience types are identical with causal role properties and the dispositionalist view that experience types are identical with dispositional properties. The impure functionalist view that experiences are essentially partly constituted by functional states and partly by categorical states also qualifies as a constitutive version of metaphysical functionalism. In contrast, the phenomenal powers view that (roughly) experiences are categorical states that are, in virtue of their character, essentially disposed to produce certain effects is a non-constitutive version of metaphysical functionalism.^34^

Let’s consider how nomological and metaphysical functionalists might explain Harmony. To explain Harmony, nomological functionalists might suppose that there are many experiential types whose instantiation conditions include the obtaining of states that would be rationalized by instances of those types. The laws might or might not mention normative phenomena. So they might or might not turn out to be teleological.

The chief difficulty with this proposal is that it runs afoul of the urgency-reduction condition. The obtaining of contingent laws that (in concert with functional states) explain Harmony is a fact that itself cries out for explanation. Again, there would presumably be a vast number of possible psychophysical laws that would not result in harmonious correlations. And it’s hard to believe that it just so happened that a set of Harmony-engendering psychophysical laws obtained in our world rather than any of the many possible non-Harmony-engendering sets of psychophysical laws. Compare: if you find it hard to believe that the fine-tuned cosmological constants obtain as a brute fact, then you will probably also find it hard to believe that they obtain because of a brutely-holding law which dictates that those constants obtain.

Metaphysical functionalists might explain Harmony by supposing that many experiential types are essentially connected either to states that are rationalized by those instances of those types or else to dispositions to cause such states. As a toy example, metaphysical functionalists might identify pain with a disposition to engage in avoidance behavior. On the assumption that there are many instantiated types of experiences that conform to this model, such theorists have an explanation of why Harmony holds: it holds because experiences just are dispositional states whose characteristic manifestations are states that they rationalize. Similarly, metaphysical functionalist might identify pain with a categorical state that is essentially disposed to generate avoidance behavior. Supposing that there are many instantiated types of experience that conform to this model, such theorists can say that Harmony holds because many experiences just are categorical states that are essentially disposed to cause certain states, states that they rationalize.

Waiving objections to metaphysical functionalism itself, the chief difficulty with these explanations is that they too violate the urgency-reduction condition. Even granting the truth of metaphysical functionalism about experiences in our world, we would expect many of the conceivable types of experiences that are not essentially connected to states their instances rationalize to be possible.^35^ (Such types of alien experiences might or might not be ones of which metaphysical functionalism is true; and they might or might not include experience types that are introspectively indistinguishable from our own.) The instantiation of these types of experience would not have underwritten harmonious correlations. These types of experience raise an urgent explanatory question for metaphysical functionalists: given all the possible types of experience whose instantiation would not explain Harmony, why are types of experience that explain Harmony instantiated in the actual world?

Metaphysical functionalists could respond by holding that the only metaphysically possible types of experience are ones whose instantiation would lend themselves to an explanation of the proposed sort for harmonious correlations. Thus, the fact that all types of experience are of this sort and that many of them are instantiated explains Harmony. In the absence of a satisfactory explanation of why all types of experience have the proposed Harmony inducing nature, this suggestion strikes me as yet another way of violating the urgency-reduction condition.^36^

### Design Hypotheses

While some theorists respond to cosmological fine-tuning by lending credence to the multiverse hypothesis, others respond by countenancing a life-favoring designer who created our universe. Similarly, biological theorists could reject panpsychism’s multitude of minds in favor of a designer who favors harmonious correlations.^37^ They could then answer my argument by rejecting Advantage and maintaining that a designer satisfactorily explains Harmony.

The main motivation for this response is straightforward: Harmony would be more likely to obtain if our universe were created by a designer who favors harmonious correlations than if it were not. There are different ways of developing this response. Some ways take the designer to select teleological laws that figure in more proximal explanations of Harmony. Others take the designer to make Harmony true by selecting a suitable form of functionalism to hold in the actual world. An occasionalist form of pre-established Harmony provides yet another option.

Of course, design hypotheses raise large questions that lie beyond the scope of any one paper. I will restrict myself to stating what I take to be the most pressing objection to combining a biological theory with a design hypothesis that is not also an objection to the design hypothesis. For the sake of argument, let’s grant biological theorists the unobvious assumption that such a designer would provide a stable, urgency-reducing explanation of Harmony, and hence that such a designer would satisfactorily explain Harmony. My objection is: if a biological theory is true, then harmonious correlations are much rarer in our universe than we would expect if it were created by a designer who favors harmonious correlations.^38^ After all, if a biological theory is true, then only a tiny fraction of physical entities are conscious, in which case at most a tiny fraction of physical entities have experiences that participate in harmonious correlations. The wasted potential for harmonious correlations in our universe coheres poorly with the hypothesis that our universe was designed to produce harmonious correlations.^39^

This might seem like a general objection to design hypotheses, rather than one geared toward preventing an unholy union between them and biological theories. But that is not so. Anyone not wedded to a biological theory who wishes to retain a design explanation of Harmony could do so by opting for *panharmonic panpsychism*. This version of panpsychism claims that while relatively few conscious physical entities are biological, most physical entities have experiences that figure in harmonious correlations. If a designer favored harmonious correlations, it seems more likely that he would create a panharmonic panpsychist world with an abundance of such correlations than that he would create a world in which a biological view of consciousness is true and harmonious correlations are relatively scarce.^40^ If so, then inferences from Harmony to a designer who favors harmonious correlations are optional detours on the way to my target conclusion that such correlations support panpsychism.

## Harmony, cosmological fine-tuning, and the total evidence requirement

My argument is reminiscent of arguments that advance cosmological fine-tuning as support for a multiverse. Such arguments start with the observation that basic physical parameters are fine-tuned in the sense that their values fall within narrow life-permitting ranges. The arguments then point out that the multiverse hypothesis that our universe is one of many in a vast and varied ensemble of universes is the best explanatory response to this observation, or that the multiverse raises the probability of the observation. Finally, it’s inferred that fine-tuning supports the multiverse hypothesis. Here’s the parallel: both sorts of argument contend that a striking outcome (Harmony and cosmological fine-tuning) supports a hypothesis on which the striking phenomena belong to a larger class of less striking phenomena.

Some theorists have argued that fine-tuning’s putative support for the multiverse hypothesis is illusory, and that arguments purporting to establish such support are fallacious.^41^ There is a large literature on this issue that I cannot engage with at length.^42^ I will restrict my focus to an objection in the multiverse case—an objection that is the same in its essentials as one pressed by White (2000)—and its extension to my argument. I focus on this objection because I take it to be initially tempting and instructively flawed.

The objection can be brought out by analogy. Suppose Winslow buys a winning lottery ticket. When he hears the winning number on the radio that night and inspects his ticket, he learns that he won the lottery. He immediately infers that someone won the lottery. Noticing that it would be more likely for someone to win the lottery if many tickets were purchased, he concludes that he has support for the *many tickets hypothesis* that many tickets were purchased.

Obviously something has gone wrong with Winslow’s reasoning. He errs in using the fact that someone wins the lottery to evaluate the many tickets hypothesis, rather than the more specific fact that he won—he flouts *the total evidence requirement* to evaluate hypotheses on the basis of one’s logically strongest relevant evidence. To respect this requirement, Winslow should have instead noticed that his winning the lottery—given *all* his relevant background information about the situation—was equally likely regardless of how many other tickets were purchased. After all, his winning the lottery just depended on his ticket number and the winning number, and these factors are independent of how many other tickets were purchased. Upon noticing that his winning was equally likely regardless of how many other tickets were purchased, he should have then concluded that his winning neither supports nor disconfirms the many tickets hypothesis.^43^

The objector holds that trying to support the multiverse hypothesis with fine-tuning for life rests on the same sort of fallacious reasoning.^44^ Granted, upon observing that our universe has constants that are fine-tuned for life we can legitimately infer that some universe has such constants. And, granted, the multiverse hypothesis raises the probability of some universe having such constants. But it doesn’t follow and it isn’t true that the multiverse hypothesis raises the probability of our universe having constants that are fine-tuned for life. Construing evidential support as probability raising, the objector concludes that our fine-tuning evidence doesn’t support the multiverse hypothesis.

The objection can be extended to my argument as follows. Here, the objection allows that we can legitimately infer Harmony from the many specific harmonious correlations that we observe. Let *Harmony*^+^ be a more specific proposition that encodes those correlations. Further, the objection allows that panpsychism raises the probability of Harmony. However, the objection maintains, it doesn’t follow and it isn’t true that panpsychism raises the probability of Harmony^+^. Panpsychism’s support from harmonious correlations therefore collapses when we take care to meet the total evidence requirement. On an explanatory (rather than probabilistic) rendering of the objection, it holds, contrary to Conditional Support, that views that can answer Harmony’s cries for explanation do not thereby enjoy support over views that cannot. For, according to the objection, it is a view’s explanatory bearing on Harmony^+^, rather than Harmony, that determines whether it is supported in this context. And while panpsychism offers a satisfactory response to Harmony, it does not offer a satisfactory response to Harmony^+^.

My initial response to this objection is that there is a crucial disanalogy between Winslow’s reasoning and the reasoning operative in both the argument for the multiverse and in my argument. Given mundane knowledge of how lotteries work, Winslow is in a position to recognize that the probability of him winning is simply 1/(the number of possible lottery ticket numbers for the lottery), regardless of how many tickets were purchased. Further, he is in a position to recognize that the method by which he acquired his evidence that someone won renders it probabilistically independent of how many tickets were purchased. Matters are different when we observe the correlations Harmony^+^ encodes or that our universe is fine-tuned. In these cases, our evidence does not include mundane background knowledge of our sampling procedures that can be used to establish that our evidence is probabilistically independent of the multiverse hypothesis and panpsychism.^45^ As a result, the objection does not show that we reach erroneous conclusions when we reason from our more general evidence to our having support for the multiverse hypothesis and panpsychism.^46^

Before considering some suggestions for resuscitating this objection, it is worth getting clear on what it takes for such objections from the total evidence requirement to show that an argument which invokes only a proper subset of our evidence fails to support its conclusion.^47^ To show that an argument violates the total evidence requirement and so falls into error, it is not enough to point out that the argument invokes only on a proper subset of our evidence. After all, we occasionally construct arguments that support their conclusions. But we virtually never reason with our total evidence. So we must have a way to support conclusions with arguments that invoke only a proper subset of our total evidence. I suggest that, for the purposes of establishing that a piece of evidence *E* supports a hypothesis *H*, reasoning with *E* is unobjectionable so long as we are not ignoring other evidence that undercuts *E*’s support for *H*.^48^ To show that fine-tuning does not support the multiverse or that Harmony does not support panpsychism, objections from the total evidence requirement must identify such an undercutter that their targets ignore. It is crucial that the identified factor undercut *E*’s support for *H*—if the factor merely disconfirms *H*, it will not have been shown that *E* does not support *H*.^49^ Thus, while aspects of fine-tuning or harmonious correlations that disconfirm the multiverse hypothesis or panpsychism may be of interest, identifying such a factor does not automatically refute the arguments under consideration.^50^

For the objection to the fine-tuning argument for the multiverse, what’s needed is a distinctive aspect of this universe being fine-tuned that prevents our evidence that some universe is fine-tuned from supporting the multiverse hypothesis. Similarly, for the objection to my argument, what’s needed is a distinctive aspect of Harmony^+^ that prevents Harmony from supporting panpsychism. Since no such factor has been supplied, the objection to my argument is at best incomplete. Let us consider three suggestions for shoring up the objection by extracting from Harmony^+^ some piece of evidence that undercuts Harmony’s support for panpsychism.

The first suggestion notes that many specific harmonious correlations encoded in Harmony^+^ are biological, i.e. they involve experiences of organisms. That Harmony has a biological realization rather than a non-biological one is a striking fact in itself, but one that is, in contrast to Harmony on its own, not rendered less striking by panpsychism. Consequently, panpsychism’s volume-reducing effect on Harmony’s explanatory cries disappears in the event that Harmony has a biological realization. Hence Harmony fails to support panpsychism after all.

There are two problems with this suggestion. One is that it’s not clear that Harmony’s having a biological realization rather than a non-biological one is a striking fact, much less that it is a striking fact that compromises panpsychism’s explanatory standing with respect to Harmony. To appreciate this point, it may help to consider a range of scenarios in which Harmony is non-biologically realized in, say, particles, molecules, rocks, stars, or galaxies. The second problem is this. While panpsychism is explanatorily idle with respect to why Harmony has a biological realization rather than a non-biological one, this does not sever the explanatory or evidential links between panpsychism and Harmony. Compare: Gary’s motives may explain and support the hypothesis that he committed the crime even if they do not explain his choice of means for carrying it out.

The second suggestion is that Harmony^+^’s featuring biological harmonious correlations undercuts Harmony’s support for panpsychism not by compromising panpsychism’s explanatory credentials but by enhancing the credentials of the biological view. After all, on panpsychism, there is no reason to expect biological harmonious correlations rather than non-biological harmonious correlations. In contrast, on the biological view, biological harmonious correlations seem much more likely than non-biological ones.

This suggestion involves a sort of bait and switch. That biological harmonious correlations are more likely on the biological view than on panpsychism would merely show that they support the biological view over panpsychism. It would not show that they undercut Harmony’s support for panpsychism. Now, since there are biological harmonious correlations and their disconfirmation of panpsychism would diminish the interest of the result that Harmony supports panpsychism, it is worth noticing that the contention that biological harmonious correlations disconfirm panpsychism is itself dubious. Admittedly, if someone’s initial evidence was just Harmony and they then learned that Harmony had a biological realization, they would thereby gain evidence for the biological view over panpsychism. However, in our own case, we possess evidence for the biological view before we come to reflect on harmonious correlations. For instance, we know that organisms have many types of experience. Evidently, the further fact that organisms have many types of harmonious experiences does not offer further support for the biological view. In treating it as support for the biological view we would be guilty of a sort of double counting.^51^

A final suggestion: Harmony^+^’s undercuts Harmony’s support for panpsychism by way of certain self-locating information, namely the fact that *our* experiences participate in harmonious correlations. While panpsychism raises the probability of Harmony if we ignore this information, it does not raise the probability of Harmony if we take this information into account. Thus, this more specific evidence undercuts Harmony’s support for panpsychism.^52^

This suggestion touches on a topic that merits further investigation: the evidential bearing of self-locating information on panpsychism.^53^ Fortunately, to address this suggestion we do not need to settle this issue. For let us grant that self-locating information tells against panpsychism. In that case, the fact that we are conscious organisms constitutes self-locating evidence for the biological view over panpsychism. Since we had this evidence in advance of considering the import of harmonious correlations, it is part of the background against which we are evaluating their import. The crucial question is whether, against this background, Harmony^+^ provides further self-locating evidence, evidence that undercuts Harmony’s support for panpsychism. Evidently, it does not. Harmonious correlations are identically distributed (but not equally striking!) among conscious organisms on the biological view and panpsychism. As a result, given that we are conscious organisms, learning that our experiences participate in harmonious correlations does not generate self-locating evidence for the biological view over panpsychism; *a fortiori*, it does not generate such evidence that undercuts Harmony’s support for panpsychism. Instead, our experiences help generate support for panpsychism by figuring in harmonious correlations that cry out for explanation to a lesser extent on panpsychism than on the biological view.

To sum up, I have explored several ways of pressing the objection that Harmony’s support for panpsychism disappears when we attend to more specific harmonious correlations. Each version of the objection was found wanting. I know of no versions of the objection that are more promising. Absent a better development of the objection, I provisionally conclude that the objection fails.

## Conclusion

Harmony is a striking datum that has been neglected in theorizing about consciousness. Panpsychism readily offers an explanatorily satisfactory place for Harmony within its conception of nature. The most promising biological responses to Harmony came up short on this score. In light of these considerations, and given that either panpsychism or the biological view is true, we should conclude that Harmony supports panpsychism. Biological theorists who wish to avoid this conclusion need to reject one of Harmony, Urgency, Advantage, or Conditional Support. Unless and until biological theorists persuasively exercise one of these options, the harmonious patterns within our conscious lives will remain a boon to panpsychism.

## Footnotes

1 For an overview of the contemporary literature on panpsychism, see Goff et al. (2017).

2 We can say that an entity is *physical* if it is posited by a fundamental physical theory or entirely composed of such entities, and *non-physical* otherwise. This definition will suffice for the purposes of this discussion, though it would need to be tweaked to avoid odd classifications in some (e.g. infinite descent) scenarios.

3 But see Godfrey-Smith (2016, p. 495), Maher (2017, Ch. 3), and Margulis (2001).

4 I adapt this move from Hawthorne & Isaacs (2018) who, following a suggestion from Cian Dorr, set aside “quasi-theist” versions of atheism that are oddly like theism in evaluating the epistemic impact of a fine-tuning argument for theism.

5 N.B. The biological view is consistent with the existence of a small number of conscious subjects that are physical but not biological. Thus, the biological view is consistent with some advanced artificial systems such as OpenAI’s GPT-3 being conscious. In the event that artificial subjects come to outnumber biological subjects, we would have grounds for rejecting the biological view that are independent of my argument. But depending on the kind of artificial subject, there may be reason to think that artificial subjects’ experiences figure in harmonious correlations no less than ours (Chalmers, 1996, Ch. 7). If so, then we could recast the argument to support panpsychism over a broadened version of the biological view that counts artificial subjects as honorary biological subjects.

6 That immaterial minds are uniquely causally paired with physical objects is a standard commitment of substance dualism—see, e.g., Foster (1991) and Swinburne (1986).

7 For a recent discussion of different forms of idealism, see Chalmers (2019).

8 There is a deep question here as to why valenced experiences give subjects such reasons and, more generally, why experiences generate the reasons that they do. Shallow answers to some instances of this question are not far to seek. For example, we can explain why pains give subjects a reason to avoid having more pains by noting that subjects have reason to avoid states that they have reason to think are bad for them and that pains give subjects reasons to think that pains are bad for them. This answer is shallow because it just pushes the question back to why pain generates *those* reasons. For a deeper answer, we would need to engage in metaethical theorizing—a task that lies beyond the scope of this paper. We need not have such an answer in hand to admit the pre-theoretical plausibility of my argument’s claims about what reasons experience generates—cf. Nagel (1986, pp. 145–146, 149).

9 There are also striking *non*-normative correlations in the vicinity of Harmony that hold between behavior and experience—cf. Crummett & Cutter (2021), Chalmers (2020), and Dalbey & Saad (2022, Section 4). These include the fact that subjects’ behavior tends to reflect magnitudes in their valence experience—for example, subjects’ pain reports tend to reflect the relative intensity of accompanying pains. This fact would be striking even if normative nihilism were true and there were no normative (quasi-)facts. This invites the question: why am I running the argument in terms of normatively harmonious correlations rather than in terms of striking phenomenal-behavioral correlations? One reason is that I think that normatively harmonious correlations contribute to the strikingness of accompanying phenomenal-behavioral correlations. For example, if normative nihilism were true, I do not think it would be as striking that pain tends to go along with avoidance behavior rather than seeking behavior. Another reason is that, as we will see, using normative terms enables a unified characterization of a broad class of correlations, a class that encompasses not only various types of striking phenomenal-behavioral correlations but also various types of striking phenomenal-phenomenal correlations. All that said, I think a variation of my argument could be run using striking phenomenal-behavioral correlations. The availability of this variation means that biological theorists cannot fully escape my argument just by endorsing normative nihilism. (Here, I am indebted to an astute referee).

10 For an overview of debates concerning cognitive phenomenology, see the introduction of Bayne and Montague (2011).

11 Harmonious correlations also play a role in the meta-problem of consciousness, roughly “the problem of explaining why we think that there is a [hard] problem of consciousness”, where the hard problem is that of explaining “why and how … physical processes in the brain give rise to conscious experience” (Chalmers, 2018, p. 6). On the plausible assumption that our experiences give us reason to think that there is a hard problem of consciousness, the meta-problem of consciousness reduces to the problem of answering Harmony’s explanatory cries within a particular domain—roughly the domain of experiences followed by judgements that lead us to conclude that there is a hard problem. See Chalmers (2020), Mørch (2020), Pautz (2020), and Saad (2019) for further discussion of harmonious correlations and the meta-problem of consciousness.

12 Goff (2018, fn 22) seems to be aware of this point.

13 Cf. Prinz (2011) and Smithies (2013).

14 For descriptions of scenarios in which experience-involving correlations are not harmonious, see Pautz (2015).

15 See White (2007, p. 455).

16 This is a stock example. I do not know its origin.

17 Cf. Leslie (1989, p. 10); see White (2000, p. 270). Plausibly, a monkey’s typing a sonnet invites a non-chancy explanation because there are non-chancy explanations of that outcome that do not have prohibitively low priors and which would render that outcome much more likely than would the chance explanation.

18 Some might be tempted to explain harmonious correlations by appealing to evolution by natural selection. However, for reasons given by Crummett and Cutter (2021) and Goff (2018, pp. 107–108), I think this strategy is a non-starter. Briefly, the trouble is that while a selectional explanation can say why—*given their causal profiles*—experiential properties are distributed in a certain way as a result of selection, such an explanation is of the wrong sort to explain why experiential properties have their causal profiles in the first place. But in asking why Harmony obtains, we are in effect asking why experiential properties have causal profiles that yield Harmony rather than causal profiles that don’t.

19 For closely related points, see Corabi (2015, pp. 212–217), Crummett and Cutter (2021), Mørch (2018, pp. 299–300), and Robinson (2007, p. 36); cf. Jackson (1982, p. 134). Those who have appealed to harmonious correlations in arguments against epiphenomenalism include Bradley (2011), Eccles and Popper (1977), and James (1890); cf. Plantinga (2004).

20 For an illuminating discussion of these criteria, see White (2005, Section 1).

21 To illustrate, suppose a thousand coin tosses come up heads. This would cry out for explanation. The hypothesis that the sequence simply resulted from fair coin tosses is unstable: such a sequence of coin tosses easily could have failed to yield the striking outcome. Intuitively, for that reason, the explanation is unsatisfactory. Note, however, that stability is not a necessary condition on satisfactory explanations in general. For example, consider instead a random-seeming sequence of heads and tails. That this sequence resulted from fair coin tosses is a satisfactory explanation. Yet it is unstable, as fair coin tosses that produced that sequence easily could have produced a different sequence instead—see *ibid*.

22 The sorts of lotteries at issue in this paper are ones in which ticket numbers and the winning number are independently selected.

23 E.g., see Lewis (1986).

24 This picture can be fleshed out in different ways, some much more plausible than others. On what I regard as an especially promising way to develop it, there is a varied ensemble of psychophysical principles that assign experiences or causal profiles to different sorts of entities—for example, maybe there is one principle assigning experiences to rocks, another assigning experiences to organisms, another assigning experiences to stars, and so on. Like other laws of nature, each of these principles is relatively simple. And since there are many of these principles and a small but not vanishingly small portion of simple psychophysical principles would induce harmonious correlations, it is to be expected that at least one of these principles will yield harmonious correlations in the kind of subject to which it applies. This way of developing the picture has the virtue of explaining why we should expect to continue observing harmonious correlations in ourselves, despite the ubiquity of non-harmonious correlations. It also reduces the extent to which Harmony cries for explanation within the macrorealm by countenancing non-harmonious correlations within some kinds of macrosubjects. While this departs from popular purely-micropsychist versions of panpsychism, I think this departure is neither surprising nor objectionable, as panpsychists have typically not used harmonious correlations as data in developing their view.

25 I owe the following objection to a referee. There is a crucial disanalogy between the ensemble of monkeys that diminishes the extent to which the monkey typing a sonnet cries out for explanation and the ensemble of minds posited by panpsychism that, according to me, diminishes the extent to which Harmony cries out for explanation: whereas the former ensemble consists of instances of the same kind (monkeys) the latter ensemble consists of instances of many different kinds. In reply, I concede that there is a disanalogy here but maintain that it is inessential. To restore the analogy, we need only suppose that, instead of there being many monkeys typing mostly random-seeming strings, there have been different kinds of animals that have been typing mostly random-seeming strings—just as that would render it less striking that the monkey types a sonnet, so too would the suggested panpsychist ensemble render Harmony less striking.

26 Functionalist, teleological, and design explanations of harmonious correlations are also considered by Goff (2018). Pautz (2015) considers a design explanation and suggests a functionalist explanation on behalf of physicalists.

27 For a defense of the coherence of fundamental teleological laws, see Hawthorne and Nolan (2006). For motivations for countenancing such laws, see Chalmers (2020), Nagel (2012), and Saad (2019, 2020a).

28 See, e.g., Kim (2005) and Papineau (2002).

29 I myself think this constraint is empirically unwarranted and I have argued that standard appeals to the causal closure of the physical to motivate rivals to interactionist dualism are misguided for other reasons (Saad, 2018, 2020b). But I agree that those views are committed to respecting the causal closure of the physical.

30 In fact, positing such a law can solve a host of problems for interactionist dualism (Saad, 2019, 2020a).

31 See Goff (2018, p. 117) for a variation of this point.

32 Another candidate pointed out by Goff (2018, p. 117): such laws obtain because of their axiological (i.e. value-involving) character. This is a version of the “axiarchic” view of the sort advanced by Leslie (1989). The problem I raise for combining a biological view with a design hypothesis in Sect. 7.3 has an obvious analog that tells against the axiarchic view. For simplicity, I will just discuss the design hypothesis in the main text. For other concerns about using an axiarchic view to handle harmonious correlations, see Pautz (2015). For further discussion of the axiarchic view, see Parfit (1998) and Rescher (2010).

33 Cf. Chalmers (1996, Chap. 7).

34 For a defense of the phenomenal powers view, see Langsam (2011, Chap. 3). For an argument for the phenomenal powers view about pain and pleasure from harmonious hedonic correlations, see Mørch (2018).

35 I’m assuming that conceivability provides defeasible evidence of possibility, whether or not conceivability entails possibility. It might be thought that metaphysical functionalists could defeat this evidence by appealing to the analytic functionalist view that the phenomenal facts of our world render it conceptually necessary that Harmony holds in any phenomenal duplicate of our world. However, this is not so. For instance, even if it is somehow conceptually necessary of the types of pain instantiated in our world that they consist partly in avoidance behavior that they rationalize, we can conceive of types of pain that are not conceptually tethered to any effects, much less to ones that they would rationalize. The conceivability of the latter types of pain is compatible with the stated analytic functionalist view. And that view provides no basis for regarding the apparent possibility of such types of pain as illusory. (Thanks to a reviewer for prompting me to consider objections from analytic functionalism).

36 I owe the following objection to a referee. That all metaphysically possible types of experience are ones whose instantiation would lend to an explanation of Harmony is a commitment of (one version of) functionalism. Since that is just part of the view, it would be illicit to charge functionalism with violating the urgency-reduction condition and failing to yield a satisfactory explanation of Harmony. In reply, that it is part of a view that it lends to an explanation of something is compatible with its failing to do so. Compare: the view that cosmological fine-tuning is a brute, metaphysically necessary coincidence and that this is a satisfactory explanation of cosmological fine-tuning is open to the charge that it fails to yield a satisfactory explanation of cosmological fine-tuning.

37 See Crummett and Cutter (2021) for a psychophysical fine-tuning argument for God.

38 For related objections to design hypotheses or attempts to support such hypotheses, see Everitt (2004, Chap. 11) and Monton (2009, p. 104).

39 Sinhababu (2017) objects to a fine-tuning argument for God by arguing that if a non-physical God has a mind, then the physical constraints on mentality are so weak that there are possible panpsychist worlds in which particles have minds like ours, in which case the conditions for minds like ours are not as stringent as the argument supposes. Sinhababu’s objection reveals a potential risk for anyone who use harmonious correlations to support theistic panharmonic panpsychism: in affirming as actual the kind of world (namely a panpsychist one) on whose possibility Sinahabu’s objection depends, they risk undermining fine-tuning arguments for theism.

40 I am evaluating this likelihood against a background that does not import religious assumptions. Against a background that includes such assumptions, matters may seem different.

41 E.g. see White (2000) and Hacking (1987).

42 For overviews, see Friederich (2021) and Manson (forthcoming).

43 Inferring that his evidence supports the many tickets hypothesis would commit the “inverse gambler’s” fallacy—see Hacking (1987).

44 See White (2000).

45 See Bradley (2009, 2012), Friederich (2021, p. 58), Juhl (2005, p. 342), Knab (2016, p. 694), and Isaacs et al. (forthcoming, pp. 4–5, p. 24).

46 I elsewhere (Saad, forthcoming) offer a positive defense of fine-tuning's support for the multiverse. While I believe that defense can be straightforwardly extended to defend Harmony's support for panpsychism, for simplicity I will not attempt such an extension or otherwise rely on that defense here.

47 I will grant the total evidence requirement for the sake of argument. But see Epstein (2017) for a challenge to that requirement and Draper (2020), Draper et al. (2007), and Barrett and Sober (2020) for defenses of the requirement.

48 Cf. Bostrom (2002, pp. 20–21). This constraint does not give us license to ignore undercutters to *E*’s support for *H*. Nor does it give us license to ignore countervailing evidence against *H* in our evaluation of *H*. Thus, the constraint is compatible with the total evidence requirement.

49 To illustrate, suppose that we find Gary’s fingerprints at the crime scene. This finding supports the hypothesis that Gary committed the crime. This is so even if we have countervailing evidence—for example, that Gary has a clean record. In contrast, if we then learned that Gary’s fingerprints were planted at the crime scene by Glenda, that would not merely disconfirm the hypothesis that Gary committed the crime; it would also undercut the fingerprint’s support for that hypothesis.

50 White (2000, p. 264) argues that, while our more general evidence (that some universe is fine-tuned) would on its own confirm the existence of other universes, we should instead reason with our more specific evidence (that *this* universe is fine-tuned), which does not confirm or raise the probability of other universes existing. However, he does not address whether the informational difference between the two bodies of evidence that explains their difference in import does so via undercutting.

51 Cf. Juhl (2007).

52 For a closely related challenge to panpsychism, see Li and Saad (2022).

53 For argument that each of the leading approaches to self-locating evidence—and any approach that meets several reasonable conditions—treats the fact that our universe is cosmologically fine-tuned as evidence for a multiverse, see Isaacs et al. (forthcoming).
